# Editorial: Factors Impacting Neural Pathways of Emotional Processing

**DOI:** 10.3389/fnbeh.2021.755286

**Published:** 2021-09-28

**Authors:** Yuval Silberman

**Affiliations:** Neural and Behavioral Sciences, College of Medicine, The Pennsylvania State University, Hershey, PA, United States

**Keywords:** editorial, emotional regulation, hippocampus, amygdala, prefrontal cortex, paraventricular thalamus, bed nucleus of the stria terminalis, diet

Emotions help guide our everyday choices in complex life situations. We are more likely to continue to perform certain tasks or to prefer certain places/situations if they induce a positive emotional valence, while avoiding those that induce negative valence. This type of emotional processing can also greatly affect memories, with emotionally salient events more likely to be remembered long-term. Such emotional processing is dependent on a widely distributed set of brain regions and neurocircuits that respond to both intrinsic and extrinsic factors. Dysregulation of these emotional processing pathways can lead to a wide variety of serious mental health disorders. Therefore, determining how intrinsic and extrinsic factors can alter emotional processing is likely to provide key understanding of the development of many mental health issues.

The goal of this article collection is to provide an update on how diverse factors, either intrinsic or extrinsic in nature, can alter neural pathways involved in emotional processing. Articles within this collection describe a number of factors that may modulate emotional regulation and result in behavioral changes. Two articles focused on the role of diets in emotional behaviors. Coker et al. reviewed the current literature examining how diets high in fat can alter key neuronal circuitries and neurotransmitter systems involved in emotional processing, how diets can alter insulin and glucose regulation, and how these seemingly disparate systems may interact in the context of alcohol use. In particular, this review focuses on the novel concept that high fat diets may impact neurocircuitry that is often associated with alcohol use disorder. Since high fat diet and alcohol intake often result in activation of overlapping circuits, this article highlights two important views: (1) binge consumption of high fat diets can sensitize increased alcohol intake and thus diet may be a risk factor for development of alcohol use disorders; and (2) high fat diets can be used to mitigate or offset neurocircuit dysregulation during alcohol withdrawal and serve to reduce alcohol intake overall. In addition to neurocircuit changes, diet and alcohol can also alter gut microbiome, which has also been shown to be involved in regulation of emotional processing. In accordance with the hypothesis that the microbiome plays an important role in altering behavioral indices of emotion regulation, Dauge et al. provided novel experimental data showing that treatment with a probiotic formula can decrease anxiety-like and depressive-like behaviors in rodent models of maternal stress and genetic stress sensitivity. Other extrinsic factors described in this collection [i.e., traumatic brain injury (McCorkle et al.), traumatic stress (Piggott et al.), and chronic drug/alcohol exposure (Dao et al.; McKendrick and Graziane)] can alter activity in brain regions known to be involved in emotional processing.

Emotional processing is thought to involve distributed networks of brain regions. In this article collection, Javanbakht et al. utilized a clinical laboratory model of fear conditioning and instructed extinction learning. Their findings indicate that subregions of the prefrontal cortex and parahippocampus gyrus show increased activation during extinction learning. Using the left ventromedial prefrontal cortex as a seed region, the authors found increased prefrontal cortex activity during extinction learning was associated with significant co-activation of the dorsal medial prefrontal cortex, parahippocampus, insula, and amygdala. Other articles in this collection utilizing preclinical models or analysis of the literature confirm the importance of the prefrontal cortex (Brockway and Crowley) and the amygdala (McCorkle et al.), and further indicate that the bed nucleus of the stria terminalis (Giardino and Pomrenze) and paraventricular nucleus of the thalamus (Barson et al.) are also critically important for regulation of emotional memories and behaviors. Within these brain regions, a number of signaling systems are proposed to be critical for emotional regulation. This is especially important in brain regions which have highly heterogenous cell-types with potential overlapping neurotransmitter and neuropeptide content, like the bed nucleus of the stria terminalis, or in brain regions like the paraventricular nucleus of the thalamus that has multiple cell types across anatomical subregions. Determining how these cell types coordinate emotional memory under typical and dysregulated conditions will be critical as the field continues to develop.

Studies to examine neuronal subtype function on behavior are underway in the prefrontal cortex, as highlighted by two studies within this collection. Both clinical and preclinical evidence suggests that a number of neuropeptides, including neuropeptide Y, corticotropin releasing factor, somatostatin, and endogenous opioids, are critical for emotional memory (Brockway and Crowley). Many of these peptides are co-released from GABAergic neurons in the prefrontal cortex and appear to be responsible for regulating the activity of both local circuits within the prefrontal cortex and the activity of glutamatergic projection neurons. Dao et al. examined how somatostatin neurons in the prefrontal cortex, basolateral and central amygdala, and bed nucleus of the stria terminalis were altered following forced abstinence from alcohol in preclinical models. This work showed that alcohol abstinence increased somatostatin neuron activity in the prefrontal cortex which may dampen overall prefrontal cortex local network activity while somatostatin neuron activity in the bed nucleus of the stria terminalis was also altered. These findings appeared to be more pronounced in female mice compared to male mice, suggesting distinct sex differences within this model. Sex differences were also shown in a study in this collection by Anderson et al. examining a subset of GABAergic neurons in the prefrontal cortex containing parvalbumin. The authors found that genetic deletion of g-protein coupled inwardly rectifying potassium (GIRK) channels from parvalbumin neurons throughout the brain resulted in increased time in the open arms of the elevated plus maze in both males and females, but an increase in immobility episodes in the forced swim test only in males. While the authors found sex differences in reversal learning models, modulation of GIRK expression in parvalbumin neurons did not seem to alter these behaviors. Modulation of GIRK expression resulted in larger afterhyperpolarization of prefrontal cortex parvalbumin containing GABAergic neurons in both sexes but this resulted in increased action potential firing only in male mice. These findings further highlight the importance of examining sex differences as the field continues to develop.

In summary, the articles of this collection highlight the complexities of understanding emotional processing. Such complexities are due to numerous factors, such as distributed neural networks, various levels of neuronal processing, and multiple neurotransmitter and neuropeptide systems. Studies in this collection describe how these factors are altered by intrinsic and extrinsic influences, such as stress, diet, or drug exposure, that can all lead to altered signaling within the distributed network of brain regions involved in emotional regulation. While future studies will be needed to further delineate such mechanisms, the articles in this collection provide novel avenues for these critically important studies to explore.

## Author Contributions

YS wrote and edited the manuscript.

## Funding

This work was supported by NIH grants AA026865 and AA022937.

## Conflict of Interest

The author declares that the research was conducted in the absence of any commercial or financial relationships that could be construed as a potential conflict of interest.

## Publisher's Note

All claims expressed in this article are solely those of the authors and do not necessarily represent those of their affiliated organizations, or those of the publisher, the editors and the reviewers. Any product that may be evaluated in this article, or claim that may be made by its manufacturer, is not guaranteed or endorsed by the publisher.

